# FuzDB: database of fuzzy complexes, a tool to develop stochastic structure-function relationships for protein complexes and higher-order assemblies

**DOI:** 10.1093/nar/gkw1019

**Published:** 2016-10-28

**Authors:** Marton Miskei, Csaba Antal, Monika Fuxreiter

**Affiliations:** MTA-DE Momentum, Laboratory of Protein Dynamics, Department of Biochemistry and Molecular Biology, University of Debrecen, H-4032 Debrecen, Hungary

## Abstract

FuzDB (http://protdyn-database.org) compiles experimentally observed fuzzy protein complexes, where intrinsic disorder (ID) is maintained upon interacting with a partner (protein, nucleic acid or small molecule) and directly impacts biological function. Entries in the database have both (i) structural evidence demonstrating the structural multiplicity or dynamic disorder of the ID region(s) in the partner bound form of the protein and (ii) *in vitro* or *in vivo* biological evidence that indicates the significance of the fuzzy region(s) in the formation, function or regulation of the assembly. Unlike the other intrinsically disordered or unfolded protein databases, FuzDB focuses on ID regions within a biological context, including higher-order assemblies and presents a detailed analysis of the structural and functional data. FuzDB also provides interpretation of experimental results to elucidate the molecular mechanisms by which fuzzy regions—classified on the basis of topology and mechanism—interfere with the structural ensembles and activity of protein assemblies. Regulatory sites generated by alternative splicing (AS) or post-translational modifications (PTMs) are also collected. By assembling all this information, FuzDB could be utilized to develop stochastic structure–function relationships for proteins and could contribute to the emergence of a new paradigm.

## INTRODUCTION

Intrinsically disordered proteins (IDPs) or protein regions (IDRs) are abundant in eukaryotic organisms and are known to serve versatile biological roles (1,2). IDPs/IDRs lack a well-defined tertiary structure in solution and exist as an ensemble of conformations in their unbound forms. The multiplicity of structures and their plasticity bias these proteins for interactions with many different partners, either simultaneously or in spatially/temporally separated fashion (3). Although the biological importance of ID proteins has been clearly established, the underlying molecular scenarios remain to be elucidated. A common assumption is that while ID proteins/regions have excessive conformational freedom in their free state in solution, this becomes rather limited when they interact with their partners (4). In other words, IDPs/IDRs adopt a well-defined structure upon recognizing a specific target, a phenomenon termed as ‘coupled folding-binding’. Increasing experimental evidence demonstrates, however, that ID proteins/regions do retain a significant degree of conformational freedom even in their partner-bound forms, exemplified by ID linkers between globular domains (5) or ID tails flanking binding motifs (6). Albeit individual cases were recognized a long time ago, the exact roles of the dynamic regions remained to be clarified. Development of modern spectroscopic techniques (e.g. NMR, FRET, single molecule approaches) (7) and ensemble characterization methods (8,9) enabled researchers to obtain deeper, higher-resolution views of dynamic regions in protein complexes. These studies demonstrated that structural multiplicity or dynamic disorder could contribute to the formation, function or regulation of the assembly, a phenomenon referred as fuzziness (10,11). Fuzzy regions not only preserve, or even increase, their conformational freedom in the bound state, but also impact the biological activity of the complex (12). Fuzziness has been shown to be a key feature in higher-order assemblies (13).

Experimental characterization of structurally heterogeneous protein complexes faces various difficulties. Fuzzy regions tend to be sticky and often result in amorphous aggregates, presenting a bottleneck for purification and impairing crystallization. The solution spectra of fuzzy complexes resembles to that found in the unbound state, often masking the actual binding sites (14). As compared to globular regions or ID regions that fold upon binding, sequences of fuzzy regions are less constrained and have looser requirements for their functions. Hence site-directed mutagenesis in fuzzy complexes may be less informative on the roles of the individual residues. Despite these obstacles, the number of experimentally observed fuzzy complexes is rapidly increasing (12).

Intrinsically disordered protein databases (Disprot (15), MobiDB (16)) collect information on ID regions of isolated proteins, which may not represent IDPs in their biological context. IDEAL (17) indicates those ID regions that undergo coupled folding and binding, based on Protein Data Bank (PDB) evidence. Those IDRs however, that remain disordered in the bound form are lacking. The Protein Ensemble Database (PED, previously known as pE-DB) (18) contains structural ensembles of ID proteins as well as their complexes, e.g. Sic1 bound to Cdc4 (19). The structural ensembles stored and analyzed in this database may provide intuitive links between the conformational characteristics and the function of IDPs. The expertise of the user in structural biology, however, strongly influences the quality of the derived relationships. Disprot also has a brief functional section, which may not be straightforward to connect to the ID state of a given region. The functional information of MobiDB (16) is imported from other databases and does not focus on the ID region(s). Similarly to the prediction-based data sets like D2P2 (20), it is suitable for larger-scale bioinformatics analyses of IDRs, but not for developing structure–function relationships.

The motivation to establish a database for fuzzy complexes is twofold. First, to collect ID proteins in their natural context, when they interact with a partner and demonstrate the functional impact of the bound ID regions. FuzDB (http://protdyn-database.org) aims to facilitate the identification and characterization of fuzzy assemblies. Second, by detailed analysis of experimental data, FuzDB relates structural heterogeneity to its biological consequences. It provides molecular insights into how fuzziness impacts the conformational ensemble and thereby its biological activity. A unique feature of FuzDB is to present the molecular scenarios and functional significance of structural multiplicity or dynamic disorder in an easily accessible format for scientists with a wide-range of expertise. In this manner, FuzDB contributes importantly to the development of a novel, stochastic structure–function paradigm (21).

## DEFINITIONS

### Criteria to define a fuzzy complex

The fuzziness of a protein assembly is evaluated on the basis of two conditions, which both have to be fulfilled.

- **Structural evidence** indicates that upon binding to a partner, a given protein region does not fold into a well-defined structure. ID regions may remain dynamic and exhibit a fast exchange of conformations in the complex state [FC0072 (22)]. Fuzzy assemblies can also be generated by ‘unfolding upon binding’ [FC0100 (23)]. Fuzzy regions do not appear in crystal structures of the protein complex, as indicated by missing electron density. For dynamic fuzzy complexes a significant degree of disorder is observed by most spectroscopic methods. As fuzzy complexes are often held together by transient interactions, their spectrum in the bound state considerably overlaps with that in the unbound state. An ID region may also alternatively fold into ensembles of structured conformations upon interacting with the same target (polymorphism) [FC0106, FC0107 (24)]. In the case of the polymorphic complexes, multiple structures of the interacting partners should be determined. Structural data for fuzzy complexes could be derived from a variety of high- (X-ray crystallography, NMR, CD, FTIR, FRET) or low-resolution (SAXS, EM, fluorescent assays, AUC, LS, AFM, MS) methods.
- **Biochemical evidence** demonstrates that the dynamic or polymorphic region impacts the formation, function or regulation of the complex. Alteration, truncation or removal of the fuzzy region modulates the binding affinity, specificity, transcriptional activity, localization or enzymatic parameters. Functional data could be derived from binding (Y2H, SPR, ITC, gel-shift), transcriptional, proteolytic or enzymatic assays as well as microscopic techniques. Sequence manipulations, such as site-directed mutagenesis, truncation or scrambling could also provide valuable functional information. Truncating fuzzy regions often results in gradual activity changes. Certain fuzzy complexes, such as yeast prions [FC0028, FC0029], acidic transcriptional activators [FC0021, FC0026] or histone tails [FC0027] tolerate a great degree of sequence modifications referred to as ‘sequence independent binding’, as long as their amino acid composition remains to be conserved.

All structural and functional techniques are listed in the **Methods** module of the database.

### Classes of fuzzy complexes

Fuzzy protein complexes can be classified on the basis of topology and mechanism. The different categories have been extensively discussed elsewhere (3,10–12); here only the most relevant points are mentioned.

Topological classes are defined based on the arrangement of the fuzzy region with reference to the globular regions or binding domains. There are two main categories: polymorphic (P), where the ID region folds into alternative structures upon binding to the same partner, and dynamic, where it interconverts between various different conformations in the complex. In dynamic complexes fuzzy regions can *flank* (F) or link—*clamp* (C)—globular binding segments. In *random* (R) complexes, short linear motifs in the fuzzy region interact with the partner in a variable manner. Assignment of topology is based on structural analysis.

Mechanistic categorization helps to decipher those molecular scenarios by which fuzzy regions interfere with protein function. Fuzzy regions may affect the conformational equilibrium of the ensemble and bias for the binding competent state (*conformational selection*) [FC0015 (25)]. They may impact the entropy of binding (*flexibility modulation*) [FC0047 (26)] or serve as an anchor to increase the local concentration of the binding site (*tethering*) [FC0060 (27)]. Fuzzy regions also may enable autoinhibitory mechanisms by intramolecular interactions established with the binding interface (*competitive binding*) [FC0052 (28)]. This information could be derived from structural analysis of the free and bound states, complemented by functional and thermodynamic data.

The different topology and mechanism categories are not exclusive; a fuzzy complex may conform to multiple classes.

## DATABASE DESCRIPTION

### Data sources and processing

**Figure 1.**
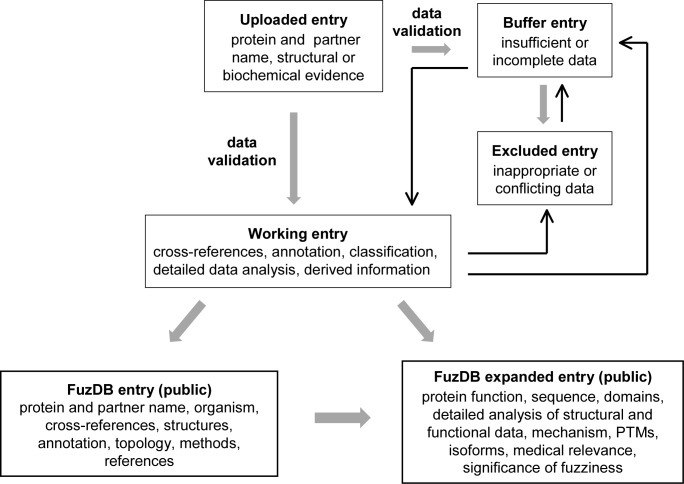
The flowchart of FuzDB data processing. Thick and thin arrows designate the default (major) and minor pathways of information exchange between the different data sets.

Data are derived from two sources: either from a literature search or uploaded via the FuzDB website (http://protdyn-database.org) (Figure 1). FuzDB has a separate module for uploading new entries that will be detailed below. The minimum information required for consideration as a database entry is the following: the name of the protein and its partner(s), and either the structural or the functional data indicating the fuzziness of the assembly. All data are manually curated and validated. The authors may be contacted directly to complement information or remove any ambiguity/concern. If the data are not sufficient to qualify for the database at this stage, the information is stored in the Buffer database. The entry is excluded if the data are not suitable or conflicting in terms of the fuzziness of the assembly. Excluded entries are also stored, as regular literature searches are performed in an attempt to find more suitable evidence. If the submitted data seem to qualify for a new entry, the information is transferred to the Working database and will be treated as a working entry. Completing information for a working entry is central to creating a new database entry in five steps (Figure 1).

- Complementing data. Cross-references are established with Uniprot, and also with the PDB and/or the Biological Magnetic Resonance Bank (BMRB) if the structure is available. Inconsistencies in sequence and organism are sorted out. Chimera sequences are marked and stored. Domain information is added based on Pfam, functional regions are indicated based on Uniprot, protein interaction databases and the literature. Post-translational modifications (PTMs), isoforms and medical relevance are collected from Uniprot and the literature. References are linked to PubMed.
- Verifying the fuzziness of the complex and defining the fuzzy region by residues. Data in the key references are corroborated by the analysis of the structure/conformational ensemble and functional data. If needed, an additional literature survey is performed, and authors may be contacted again. If the data are still incomplete, the working entry is transferred to the Buffer database. Entries can be freely exchanged between the Working and the Buffer databases. Entries may also be excluded from the Working database in the case of conflicting data.
- Classification of the fuzzy region based on topology and mechanism.
- Detailed structural and functional analysis. Information is extracted from the references and is compiled in a concise manner. The goal is to highlight the type of structural information that is indicative of fuzziness and also its functional relevance in a very clear, possibly quantitative, way. Two descriptive sections are formulated and added to the working entry.
- Deriving structure-function relationships. If data are available, the mechanism by which the fuzzy region impacts the conformation ensemble and thereby the biological activity of the assembly is detailed. The significance of fuzziness is highlighted.

The completed working entry is transferred to the publicly accessible FuzDB. The minimum information needed for a public FuzDB entry is the following: the name of the protein and its partner, cross-reference to Uniprot, protein function, both structural and functional data supporting fuzziness, detection method, annotation and classification of the fuzzy region, significance of fuzziness and key reference(s). Additional data can be incorporated at later stage via the Working database. During the updating process, the original public FuzDB information remains to be displayed. The public FuzDB entry is presented in two parts, in the **Table of Entries** and in the corresponding **Expanded Entry** page, as will be detailed below.

### Database organization

FuzDB has a modular architecture. The **Home** module introduces fuzziness and the criteria to define fuzziness based on the cited reviews. The **Browse** module displays the **Table of Entries** of 108 fuzzy complexes grouped into 10 complexes per page. This contains the core data, and each complex also has an associated **Expanded Entry** page, where the detailed analysis and derived information is presented. The **Search** module enables a simple text or advanced search across the database. Specific terms related to derived information, such as the mechanism category, can also be used. The **Methods** module collects the experimental techniques that have been applied to study fuzzy assemblies. **Methods** is linked to the Detection method column of the **Table of Entries** in the **Browse** module and can also appear as a pop-up window. The **Classes** module describes the topological and mechanistic categories of fuzzy complexes. This is linked to the Class column of the **Table of Entries** in the **Browse** module and can also appear as a pop-up window. The **Upload** module is for submission of new entries to the database. This module is highly protected, requiring a safety key to upload data, which can be obtained by email. The process of submission is described in detail step-by-step and the data will be validated (see above). The **Analysis** and **Help** modules provide information on how to use the resource. The Analysis module details examples on how to interpret data for individual systems and how to perform comparative analysis of fuzzy complexes. The **Help** module describes the modules as well as the columns in the **Table of Entries** and the fields in the **Expanded Entry** page. The **FAQs** module discusses questions related to conceptual issues and biological significance of fuzzy complexes.

### Table of entries

**Figure 2.**
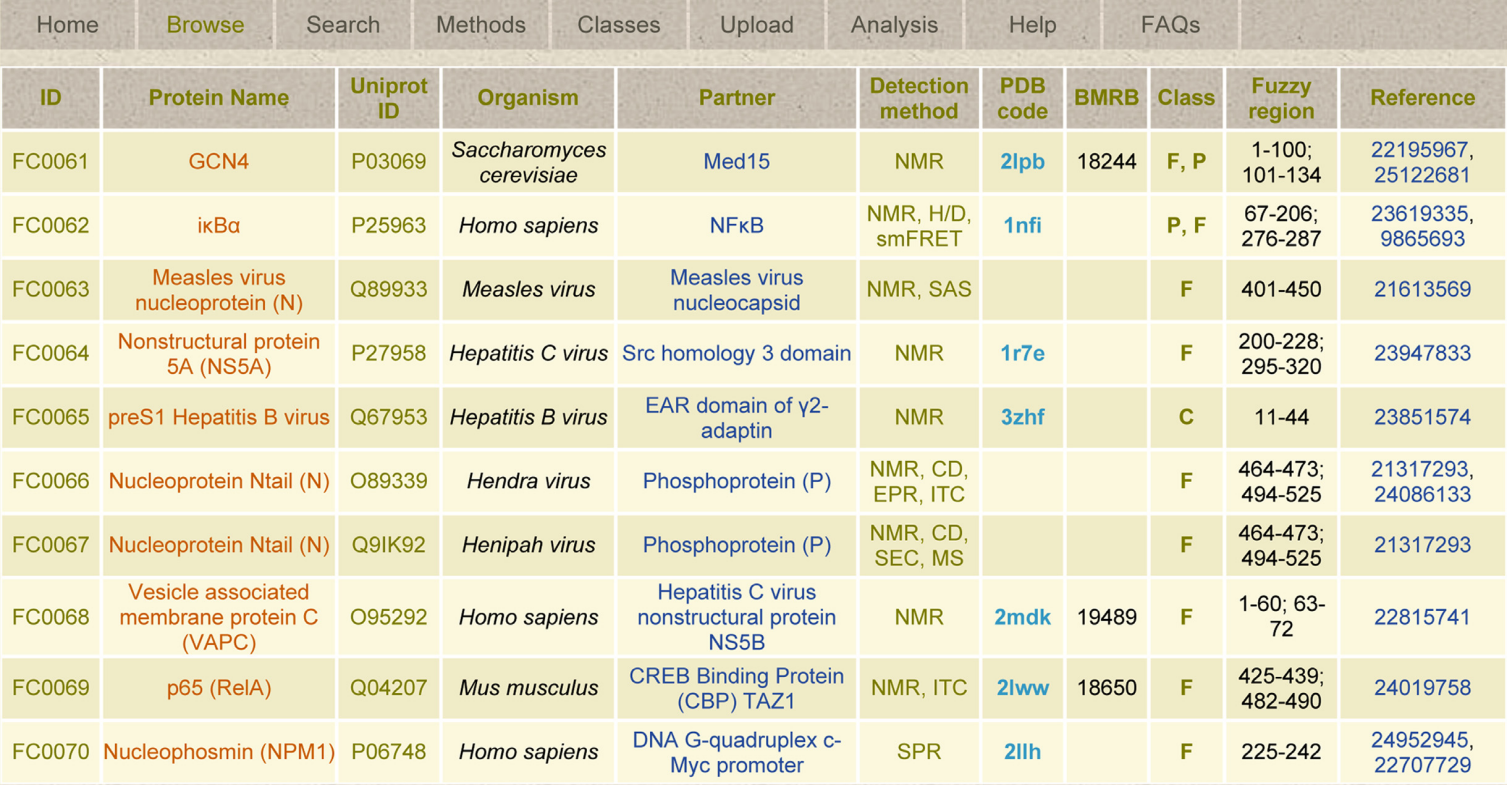
The FuzDB modules and the **Table of Entries** of the **Browse** module.

The core of FuzDB is the **Browse** module (Figure 2). Data in the **Table of Entries** provides basic information about the assembly, including the specification of the partners and the annotation of the fuzzy region(s). All fuzzy complexes have an identifier in the format of FCxxxx, and the same protein can be involved in multiple entries. The protein name also includes domain specifications and abbreviations. The Uniprot identifier is defined for the ID protein and links to the sequence in fasta format. In the case of chimeras, the sequence is displayed in the **Expanded Entry** page. The organism of the protein is also reported in the **Table of Entries**. If the structure is available, a cross-reference to the Protein Data Bank and/or the Biological Magnetic Resonance Bank is provided. The Detection method(s) column lists the experimental techniques and is linked to the **Methods** module. Fuzzy regions are annotated by defining the start and terminating amino acids with reference to the Uniprot sequence; multiple regions are separated by colons. The topological classification of the fuzzy region is displayed in the Class column, which is linked to the **Classes** module. PubMed identifier(s) of the key reference(s) is/are given in the last column. Detailed information on each complex can be found in the corresponding **Expanded Entry** page by clicking either the FCxxxx identifier or the protein name.

### Expanded entry page

**Figure 3.**
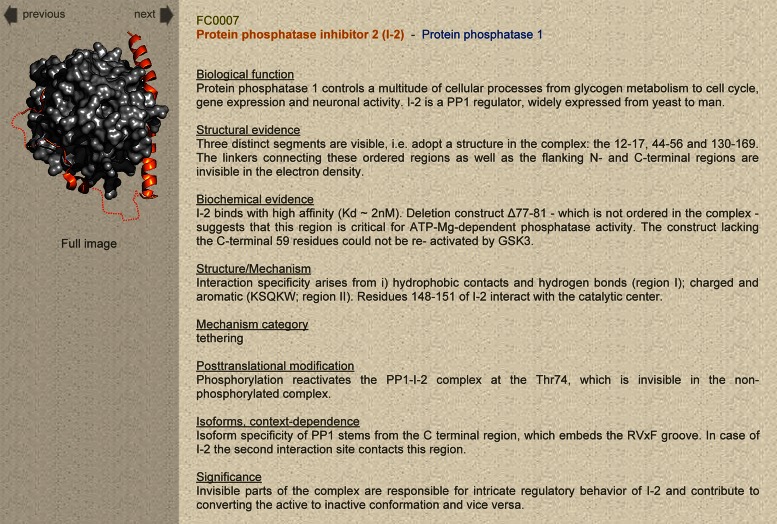
FuzDB Expanded Entry page.

The **Expanded Entry** page provides detailed insights into the experimental characterization of fuzzy complexes and higher-order assemblies, and can be used to develop structure-function relationships. If coordinates are available, the structure is displayed on the left side with an option to view the full, higher-resolution image (Figure 3). Fuzzy regions are marked by dotted lines, and arrows above the model allow the user to switch between the different entries. The right side of the **Expanded Entry** page contains information related to the structure, function and mechanism of the assembly (Table 1). Information on domains, protein function and medical relevance is compiled based on other databases (Uniprot, Pfam, Cosmic) and the literature. Post-translational modification sites or alternative splicing, which affect the fuzzy region, are also listed together with their functional relevance.

**Table 1.** Description of fields in the **Expanded Entry** page

Field	Description	Source
Biological function	Protein/assembly function(s) with specification to the fuzzy region.	Uniprot, PubMed*
Domain organization/ sequence features	Topological organization of the protein, ID regions and domains. Main sequence features. Chimera sequences.	Pfam, Uniprot, PubMed
Structural evidence	Structural data demonstrating structural multiplicity or dynamic disorder in the complex.	PDB, BMRB, references
Biochemical evidence	Functional data demonstrating the impact of the fuzzy region in the formation, activity or regulation of the assembly.	references
Structure/mechanism	Molecular mechanism of how fuzzy region(s) modulate the conformational ensemble and the function of the assembly.	derived information, references
Mechanism category	Mechanistic classification of the fuzzy region.	derived information
Post-translational modification	The role of PTMs interfering with the fuzzy region and their impact on the activity of the complex.	Uniprot, references, derived information
Isoforms, context-dependence	Different isoforms, generated by alternative splicing affecting the fuzzy region. Functional and context-specific features of the different isoforms.	Uniprot, references, derived information
Medical relevance	Involvement in different diseases, specific roles of the fuzzy regions.	Uniprot, Cosmic, literature
Significance	The relationship between fuzziness and the function of the assembly.	derived information
Further reading	Additional references.	PubMed

*PubMed means a general literature search, references refer to the articles specified in FuzDB.

The **Expanded Entry** page presents a detailed structural and functional characterization of the fuzzy complex. The structural data provide insights into those features that could be observed by a given experimental technique, e.g. timescales of motions of the fuzzy region. Functional data include quantitative results, such as affinity, transcriptional activity, catalytic rates or half-life values obtained on different variants, where the fuzzy region is affected. A unique feature of FuzDB is that it also gives derived information such as the mode of action of the fuzzy region or the biological relevance of fuzziness. The mechanism by which the fuzzy region impacts the function of the complex is based on the interpretation of the structural data or model and is classified into four categories. Highlighting the significance of fuzziness establishes the link between the structural heterogeneity of a given region and the biological activity of the complex. The **Expanded Entry** page (Table 1) presents 11 sections, of which the biological function, structural and biochemical evidence, mechanism category and significance of the fuzziness are obligatory, the others depend on the experimental data available.

## USING FuzDB

### Searching FuzDB

In the **Search** module the user may perform a *simple text* and an *advanced* search to find entries in the database; both are case insensitive. The search results can be downloaded using the ‘Text view’ option in the right side of the menu. The *simple text* search is not limited to specific columns/fields, all sections of the **Table of Entries** and the **Expanded Entry** are reviewed. A *simple text* search is an efficient way to look for expressions that are located in the descriptive sections of the **Expanded Entry** page. For example, applying the ‘unfolding’ filter will select proteins that unfold or expand upon binding to their partners. This option is also useful to collect assemblies with specific functions or to list fuzzy complexes from a set of species (e.g. from yeasts, using Saccharomyces).

The *advanced search* is recommended when looking for terms in specific sections, such as protein or partner name, organism, the PDB or BMRB code, Uniprot or PubMed ID. The user may also search for fuzzy complexes that have been determined by a given experimental technique, as listed in the **Methods** module. It is possible to use a combination of specific fields, such as topology and mechanism. For example, applying ‘flanking’ (topology) and ‘tethering’ (mechanism) filters will select those fuzzy regions that flank ordered binding sites and serve to anchor them to their partners [FC0064 (29)]. In this manner the Search module can also be used for comparative analysis of fuzzy complexes belonging to different categories.

### Uploading to FuzDB

Information submitted by users is a valuable source to expand the database. At present only new entries can be submitted; additional information to existing entries should be sent by email and not via the website. The submission process is described step-by-step in the front page of the **Upload** module. By providing an email address, the user receives an electronic code that enables him/her to submit data. The required fields are the name of the protein and partner(s) and either the structural or the biochemical evidence, although for a new FuzDB entry both types of data will be required. The user is advised to provide a reference, such as a PubMed or a structure identifier. Specifying the sequence of the protein is recommended, but is not obligatory. An automatic email is generated upon submission to notify the FuzDB team. In most cases, the authors are contacted for missing or ambiguous information and an extensive literature search is performed to complement data (see Data Processing). Obviously, email inquiries regarding fuzzy complex candidates are also considered. The contact name and email of the submitter is displayed in the bottom of the **Expanded Entry** page.

### Analysis of fuzzy complexes

FuzDB can be used to perform comparative analysis or to interpret experimental data for the system of interest, as exemplified in the Analysis module. In the case of a fuzzy complex candidate, a technique-specific filter can be applied in the *advanced search* of the **Search** module. Fuzzy complexes, which have been studied by the given experimental approach, will be selected and their corresponding **Expanded Entry** pages detail the structural evidence indicating dynamic disorder or polymorphism in the bound state. For functional analysis, proteins with related activities can be selected in the *simple text search* of the **Search** module, and their corresponding **Expanded Entry** pages discuss the *in vitro* or *in vivo* biochemical data supporting fuzziness. Once both criteria are fulfilled and the fuzziness of the assembly is confirmed, it is advisable to classify the complex based on topology. Then the topology class filter in the *advanced search* of the **Search** module can be applied to relate the complex to other assemblies of the same class.

A comparative analysis can be performed via the **Search** module on data either in the **Table of Entries** or the **Expanded Entry** page, by organism, partner, function, topology or mechanism. Individual or combined categories can be used, e.g. comparing different topology classes in the same organism or analyzing mechanisms derived from the same experimental approach. The data and cross-links can be subjected to bioinformatics analysis using more advanced computational tools, e.g. to quantify the propensity of fuzzy region(s) within a protein or to compare the lengths of fuzzy regions within an organism.

### Deriving structure-function relationships and regulatory mechanisms

One of the goals of FuzDB is to provide not only data, but also derived information on the relationships between structural ensembles and biological activities. The molecular scenarios by which fuzzy regions affect conformational ensembles and influence functions are discussed in the **Expanded Entry** pages. These can be related to the observations on the system of interest, or can be related to other assemblies, either from the same organism, function or topological class, or those studied using the same experimental approach.

Regulation of protein assemblies often involves fuzzy regions (11,13) that can be targeted by post-translational modifications or may be subjected to alternative splicing. The PTMs and the different isoforms may activate/inhibit a binding motif, which is located in the fuzzy region, or fine-tune its interactions with a specific partner. These effects can be of crucial importance in a variety of biochemical processes, e.g. cell-cycle regulation, or may have the utmost medical relevance, such as in the case of c-Myc (30). An overview of regulatory features of the fuzzy region is given in the *post-translational modifications* and the *isoforms, context-dependence* sections of the **Expanded Entry** page. Context-dependence refers to environment-dependent functional features of the different isoform, which are modulated by the PTM or AS of the fuzzy region. This information can complement and be combined with the molecular mechanism that is described in the **Expanded Entry** page, and can help to interpret the structural and functional properties of the system of interest under different conditions.

## CONCLUSIONS AND PERSPECTIVES

Structural biology is engraved in a deterministic relationship between protein sequence, structure and function. Proteins without a well-defined three dimensional conformation in their native state contradict this paradigm and transform our thinking on the structural pre-requisites of biological functions. Upon interacting with their partners, however, ID proteins were assumed to adopt a folded state and still follow the classic principles. In contrast, the concept of fuzzy complexes establishes a direct relationship between the structural heterogeneity and biological function of proteins within their physiological context. Conformational freedom is not only preserved in fuzzy assemblies, but also contributes to their interactions, activities or regulation.

FuzDB collects fuzzy assemblies of proteins and provides annotations for fuzzy protein regions. It contains proteins with a wide variety of functions, ranging from gene expression regulation, signaling, endocytosis, aggregation, chaperoning, cytoskeleton organization, as well as enzymes (12), which may not be present in more conventional ID-related databases. All data are manually curated and importantly, validated. In addition, FuzDB provides detailed structural and functional evidence for fuzziness, as well as molecular mechanisms for deriving structure–function relationships.

All entries in FuzDB are based on experimental data. An ongoing effort is to develop a computational approach to predict fuzzy regions that tend to remain unfolded even when bound. This will be an efficient engine to identify further candidates, which will be validated on the basis of experimental results. Preliminary studies indicate ∼5000 long fuzzy regions (>30 AA) in the human proteome (M. Miskei, M. Fuxreiter in preparation). The prediction algorithm for fuzzy regions will be incorporated in a web-based server and will be linked to FuzDB. Predicting fuzzy regions facilitates pre-screening of new candidates and promotes analysis of biologically important complexes.

In summary, FuzDB facilitates the identification of fuzzy protein assemblies and establishes a direct connection between the conformational ensembles and functions of proteins. This is the basis of a new, stochastic paradigm, leading to a more holistic view on how proteins fulfill their biological roles.
